# Cannabidiol reduces LPS-induced inflammatory response in the human placenta by reducing NF-κB translocation

**DOI:** 10.1186/s42238-025-00369-6

**Published:** 2025-12-06

**Authors:** Ramon Portillo, Tetiana Synova, Mohammad Rida Ghaddar, Mia Salma Alsouki, Fiona Kumnova, Miloslav Machacek, Rona Karahoda, Cilia Abad, Frantisek Staud

**Affiliations:** 1 Department of Pharmacology and Toxicology, Charles University, Faculty of Pharmacy in Hradec Kralove, Akademika Heyrovskeho 1203, Hradec Kralove, 500 05 Czech Republic; 2 Department of Biochemical Sciences, Charles University, Faculty of Pharmacy in Hradec Kralove, Akademika Heyrovskeho 1203, Hradec Kralove, Czech Republic

**Keywords:** Cannabidiol, Placenta, Explants, Inflammation, Trophoblast, Cytokine, Lipopolysaccharide, NF-κB, Immune modulation

## Abstract

**Background:**

Cannabidiol (CBD), a non-psychoactive cannabinoid increasingly used during pregnancy, has been proposed to modulate inflammatory processes. However, its effects on human placental immune functions remain poorly characterized. This study investigates the impact of CBD on lipopolysaccharide (LPS)-induced inflammation in human placenta explants and primary trophoblast cells, focusing on cytokine expression, receptor involvement, and underlying mechanisms.

**Methods:**

Term placental explants and syncytiotrophoblast cells were exposed to LPS with or without CBD. Inflammatory cytokine levels were quantified by ELISA and RT-qPCR. Receptor involvement was assessed using selective antagonists for cannabinoid receptors type 1 and 2 (CB1 and CB2), and transient receptor potential cation channel subfamily V member 1 (TRPV1). NF-κB activation was evaluated by immunofluorescence, and caspase-1 activity was measured to explore inflammasome-related pathways.

**Results:**

CBD significantly attenuated LPS-induced interleukin-1β (IL-1β), tumor necrosis factor alpha (TNF-α), interleukin 6 (IL-6), and interleukin 18 (IL-18) expression in a concentration-dependent manner, without inducing cytotoxicity. These effects were not reversed by CB1, CB2, or TRPV1 antagonists, indicating that other pathways are likely involved. CBD was associated with reduced NF-κB p65 nuclear translocation yet did not affect caspase-1 activity or transcript levels, indicating inflammasome-independent suppression.

**Conclusion:**

CBD exerts anti-inflammatory effects in human placenta and trophoblasts, associated with reduced NF-κB p65 nuclear translocation and independent of CB1, CB2, and TRPV1 signaling, without evidence of canonical inflammasome activation. Given the placenta’s role in fetal programming, these findings underscore the importance of evaluating CBD's developmental impact in the context of its growing perinatal use.

**Graphical Abstract:**

Graphical abstract illustrating the proposed mechanism by which CBD attenuates LPS-induced inflammation in human placental explants and primary trophoblast cells. LPS promotes NF-κB p65 nuclear translocation and increases expression of IL-6, IL-1β, and TNF-α. CBD reduces NF-κB nuclear accumulation and downstream cytokine production. Dotted trajectories denote hypothetical or unresolved intermediate signaling steps and do not imply direct molecular interactions. Functional antagonist assays indicate that CBD-mediated immunomodulation in the placenta occurs independently of CB1, CB2, and TRPV1 receptors. This schematic is presented as a working hypothesis. Created with BioRender.com.

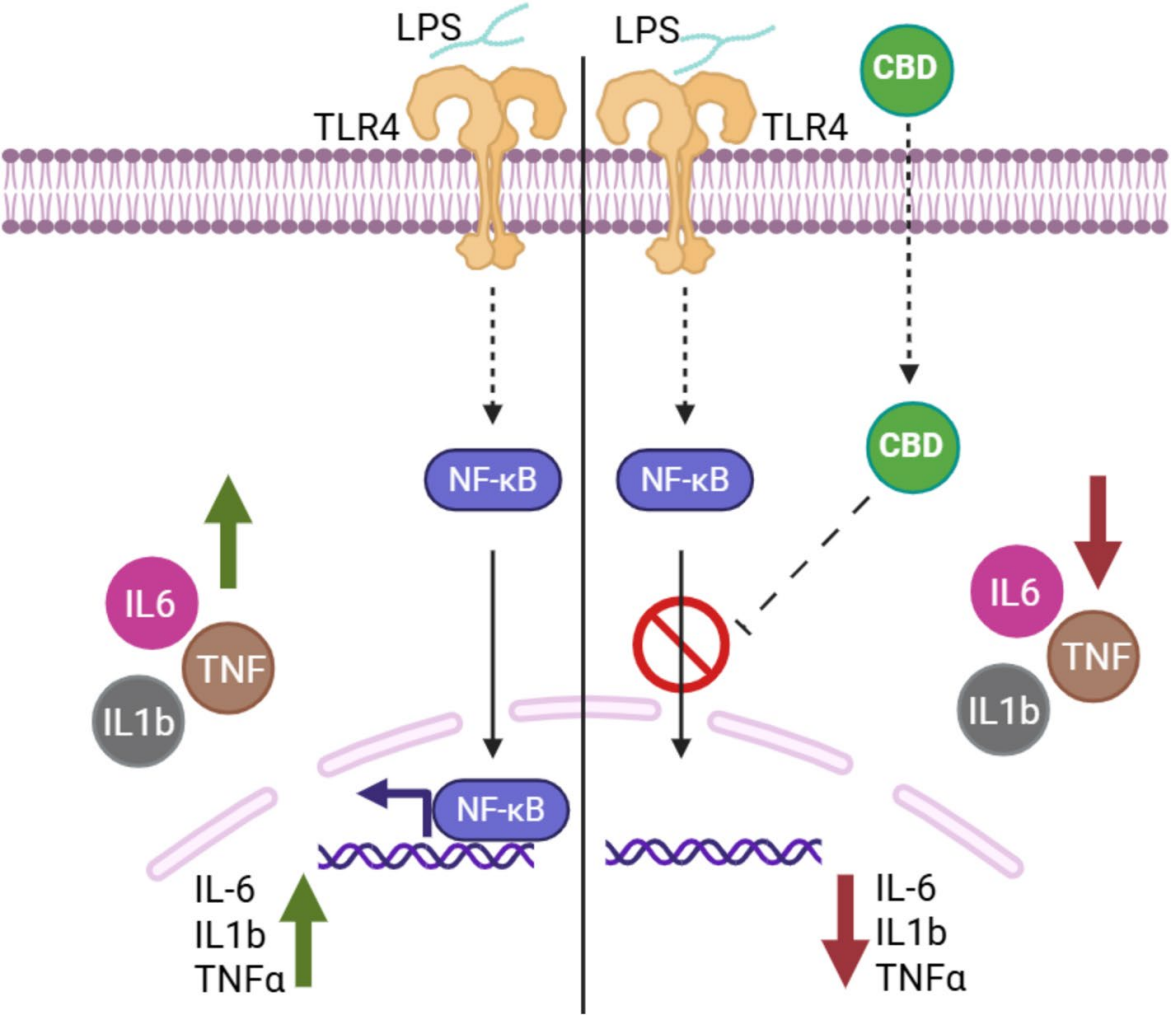

**Supplementary Information:**

The online version contains supplementary material available at 10.1186/s42238-025-00369-6.

## Introduction

Pregnancy depends on a delicate, finely tuned immunological balance between pro-inflammatory and anti-inflammatory processes. This dynamic equilibrium, orchestrated by hormonal fluctuations and intricate cytokine signaling pathways, is critical not only for successful implantation but also for the proper development of the placenta and healthy fetal growth (Meyyazhagan et al. 2023). Disruptions in this balance can lead to adverse outcomes such as preeclampsia, preterm birth, fetal growth restriction, and long-term developmental issues, as outlined by the Developmental Origins of Health and Disease (DOHaD) theory (Barker 2004). The placenta, serving as the vital interface between maternal and fetal systems, regulates immune responses to maintain fetal homeostasis by modulating maternal immune tolerance and protecting the fetus from pathogens and inflammation. However, its functions are vulnerable to inflammatory insults and infections, which can disrupt this immune balance, impair trophoblast function, and jeopardize both placental and fetal health​ (Ding et al. 2022). Given the critical role of immune balance in pregnancy, external factors that influence inflammatory pathways—such as bioactive compounds—can have significant implications for both maternal and fetal health. One such compound is cannabidiol (CBD), a natural cannabinoid found in *Cannabis sativa*, which is gaining popularity among pregnant women due to its purported therapeutic properties (Gesterling and Bradford 2022).

The global rise in cannabis use, driven by evolving legislation and shifting societal attitudes, has raised significant public health concerns (Chiu et al. 2021). Phytocannabinoids are characterized by complex pleiotropic pharmacology (Blebea et al. 2024), with CBD representing a particularly intricate example that exerts anxiolytic, antiemetic, and anti-inflammatory effects through interactions with the endocannabinoid system (ECS) and additional molecular pathways (Britch et al. 2021)​. Pregnant women are increasingly using CBD to manage symptoms such as nausea, anxiety, and inflammation, often perceiving it as a safer alternative to conventional medications. While CBD and THC demonstrate antiemetic effects in chemotherapy-induced nausea, their mechanisms and long-term safety profiles remain unclear, and paradoxical effects, such as cannabinoid hyperemesis syndrome, complicate their clinical use (Bathula and Maciver 2023; Loganathan et al. 2024). Despite its growing popularity, the scarcity of safety data, coupled with emerging evidence linking even short-term CBD exposure in placental models to disruption of placental and trophoblast functions raises significant concerns about its use during pregnancy (Martin 2020; Roussos-Ross et al. 2022; Portillo et al. 2024; Alves et al. 2021; Rokeby et al. 2023; Berman et al. 2023).

CBD does not act as a direct agonist or antagonist at CB1 or CB2 receptors. Instead, CBD exhibits low orthosteric affinity at these receptors, can negatively modulate CB1 allosterically, and antagonize CB1/CB2 agonist signaling in vitro. CBD may also alter endocannabinoid tone indirectly (e.g., by inhibiting FAAH) and engages non-cannabinoid targets, including 5-HT1A, TRPV1, GPR55, adenosine receptors, and PPARγ (Almeida and Devi 2020). It modulates immune responses, suppressing pro-inflammatory cytokines and promoting anti-inflammatory pathways (Martinez Naya et al. 2024). However, emerging evidence suggests that the biological context plays a crucial role in determining whether cannabinoids act as anti-inflammatory or pro-inflammatory agents (Henshaw et al. 2021). In the context of pregnancy, the dual nature of inflammation—as both a necessary physiological process and a potential pathogenic mechanism (Brann et al. 2019)—raises concerns about the use of CBD. Aberrant inflammation in the placenta is known to contribute to adverse pregnancy outcomes, yet the effects of CBD on these processes, and its potential interactions with ECS receptors and key inflammatory pathways, remain largely unexplored.

This study investigates the effects of CBD on lipopolysaccharide (LPS)-induced inflammation in human placental explants and in primary human trophoblasts differentiated to the syncytiotrophoblast (STB) stage. Specifically, we assess whether CBD influences pro-inflammatory cytokine production and gene expression, and whether its actions involve CB1, CB2, and TRPV1 receptors. Additionally, we explore the potential mechanisms underlying CBD’s immunomodulatory properties. By elucidating these mechanisms, this research aims to provide evidence-based insights into the effects of CBD use during pregnancy.

## Methods

### Chemicals and solutions

CBD (purity ≥ 98.5%, HPLC) was purchased as a 10 mg/mL ethanol-based stock solution from Sigma-Aldrich (St. Louis, MO, USA). Prior to each experiment, CBD was diluted directly into culture medium to the desired final concentrations, ensuring that ethanol content did not exceed 0.4% (v/v). The receptor ligands used for mechanistic assays, rimonabant (CB1 inverse agonist), SR144528 (CB2 inverse agonist), and SB366791 (TRPV1 antagonist), were also obtained from Sigma-Aldrich and prepared at a final concentration of 10 μM in culture medium. LPS (*Escherichia coli* O111:B4) was acquired from Sigma-Aldrich and freshly dissolved in culture medium prior to application.

### Placenta collection

Human placentas were obtained from healthy full-term pregnancies (gestational age 38–40 weeks) of women undergoing elective cesarean sections at the Department of Obstetrics and Gynecology, University Hospital Hradec Kralove, Czech Republic. The placentas were immediately transported to the laboratory for subsequent analysis. All procedures adhered to the ethical standards outlined in the Declaration of Helsinki. Participants provided written informed consent, and the study was approved by the Research Ethics Committee of the University Hospital (201006 S15P).

### Human placenta explant culture

Human term placentas (*n* = 10) were visually inspected, and regions showing signs of calcification or visible damage were excised before further processing. Individual cotyledons were dissected from various regions, with decidua basalis and the chorionic plate carefully removed. The remaining villous tissue was rinsed in cold saline to eliminate excess blood, then trimmed into individual explants weighing 30 ± 5 mg, discarding large vessels and blood clots. Approximately 100 mg of tissue was placed into each well of a 12-well culture plate. Placental explants were cultured in Dulbecco's Modified Eagle Medium/Nutrient Mixture F-12 (DMEM/F-12) containing 10% fetal bovine serum (Capricorn Scientific GmbH, Germany), and a cocktail of antibiotics and antimycotics (100 U/mL penicillin, 0.1 mg/mL streptomycin, 2.5 µg/mL amphotericin B) (Sigma-Aldrich) at 37 °C in an atmosphere of 8% O₂, 5% CO₂, and 87% N₂. Following an 18–24-h stabilization period, explants were treated with CBD (7.95, 31.80, 63.60, or 127.20 µM) for 48 h, with the culture medium replaced every 24 h. Subsequently, explants were exposed to an additional 4-h treatment with 1 µg/mL LPS combined with the respective concentration of CBD. At the end of the experiment, placental explants were gently rinsed in cold sterile saline, weighed, and collected for downstream analyses. Culture supernatants were centrifuged at 10,000 × g for 15 min to remove debris, and both explant tissues and clarified media were stored at − 80 °C until further processing.

### Primary trophoblast cell isolation

Primary trophoblast cells were isolated from term human placentas (*n* = 6) based on a previously described protocol (Huang et al. 2016; Karahoda et al. 2020). Briefly, ~ 300 g of placental tissue was dissected, washed, and cleaned to yield ~ 30 g of villous tissue. Three successive 30-min digestions were performed at 37 °C in 0.25% trypsin (Gibco, Thermo Fisher Scientific, Waltham, MA, USA) and 300 IU/ml DNase I (Sigma-Aldrich). Cytotrophoblast cells were separated using Percoll® density gradient (Cytiva, Marlborough, MA, USA). Purity and characterization of isolated primary trophoblast cells were assessed via flow cytometry. Cells were fixed and permeabilized, then incubated on ice for 1 h, protected from light, with a panel of antibodies specific to trophoblasts and to common non-trophoblast contaminants. The following antibodies were used: anti–cytokeratin 7 (Alexa Fluor 488®) for trophoblasts, anti–von Willebrand factor (Alexa Fluor 647®) for endothelial cells, and anti-vimentin (Alexa Fluor 647®) for stromal cells (all from Novus Biologicals, Centennial, CO, USA) (Karahoda et al. 2020). Flow cytometry was performed using an SA3800 Spectral Cell Analyzer (Sony Biotechnology, USA), and data from at least 10,000 events were recorded per sample with FCS Express Software (De Novo Software, USA). Only preparations with a purity greater than 90% were included in subsequent experiments.

### Primary trophoblast cell treatment experimental conditions

Primary trophoblast cells were seeded into 24-well plates at a density of 6.0 × 10^5^ cells per well and incubated at 37 °C in a humidified atmosphere of 5% CO₂ and 95% air. Cells were maintained in High-glucose DMEM with GlutaMAX™ (Gibco, Thermo Fisher Scientific) supplemented with 10% fetal bovine serum and penicillin/streptomycin at a final concentration of 100 U/mL and 100 µg/mL, respectively (Sigma-Aldrich). Primary human trophoblasts were cultured for 72 h to allow spontaneous syncytialization to STB, with daily medium changes (Vachalova et al. 2022).

Following this pre-incubation, syncytiotrophoblasts were subjected to one of two experimental protocols:

(1) CBD alone, or (2) CBD in combination with selective receptor antagonists.

1. CBD-only exposure: Cells were treated for 24 h with CBD at concentrations of 0.32, 3.18, 15.90, or 31.80 µM. This was followed by a 4-h incubation with fresh medium containing the same concentration of CBD and 0.5 µg/mL LPS to induce an inflammatory response.
2. CBD combined with receptor antagonists: To explore potential receptor pathways mediating the anti-inflammatory effect, cells were pre-incubated for 30 min with one of the selective antagonists: rimonabant (CB1 inverse agonist, 10 µM), SR144528 (CB2 antagonist, 10 µM), or SB366791 (TRPV1 antagonist, 10 µM). Cells were then co-treated for 24 h with CBD (0.32, 3.18, 15.90, or 31.80 µM) and the respective antagonist, followed by a 4-h stimulation with LPS (0.5 µg/mL), with continued exposure to both CBD and the antagonist.

At the end of the treatments, culture media were collected and centrifuged at 10,000 × g for 15 min to remove cellular debris. Supernatants were stored at − 80 °C for subsequent analyses. For RNA isolation, cells were lysed in-well using TRI Reagent® (Molecular Research Center, Cincinnati, OH, USA), with gentle agitation to ensure complete lysis prior to sample collection.

### RNA isolation, reverse transcription, and RT-qPCR analysis

Total RNA was extracted from either STB-stage primary trophoblasts or approximately 100 mg of human placental explant tissue using TRI Reagent® (Molecular Research Center), in accordance with the manufacturer’s instructions. RNA quantity and quality were assessed spectrophotometrically using the NanoDrop™ 1000 system (Thermo Fisher Scientific). The RNA concentration was calculated based on absorbance at 260 nm, while purity was verified by evaluating A260/A280 and A260/A230 ratios to exclude protein or solvent contamination. First-strand cDNA synthesis was performed using the iScript™ Advanced cDNA Synthesis Kit (Bio-Rad Laboratories, USA) on a T100™ Thermal Cycler, following the standard protocol provided by the supplier. Resulting cDNA was diluted to a working concentration of 12.5 ng/µL for downstream amplification. Quantitative real-time PCR (RT-qPCR) was carried out using a QuantStudio™ 5 system with TaqMan® Gene Expression Assays (Thermo Fisher Scientific). Each 5 µL reaction contained 1 µL of diluted cDNA, 0.25 µL of a gene-specific TaqMan assay, 2.5 µL of TaqMan® Fast Advanced Master Mix (no UNG; Thermo Fisher Scientific), and 1.25 µL of nuclease-free water. Amplification was performed using the following thermal cycling conditions: initial denaturation at 95 °C for 20 s, followed by 40 cycles of 95 °C for 1 s and 60 °C for 20 s. All reactions were performed in technical triplicate.

For placental explants/cells, the following genes were used: NLR family pyrin domain containing 3 (*NLRP3;* Hs00918082_m1), caspase 1 (*CASP1;* Hs00354836_m1), interleukin 18 (*IL18;* Hs01038788_m1), toll like receptor 4 (*TLR4;* Hs00152939_m1), nuclear factor kappa B subunit 1 (*NFKB1;* Hs00765730_m1), interleukin 6 (*IL6;* Hs00174131_m1), interleukin 1 beta (*IL1B;* Hs01555410_m1), tumor necrosis factor (*TNF;* Hs00174128_m1), peroxisome proliferator activated receptor gamma (*PPARG;* Hs01115513_m1), C-X-C motif chemokine ligand 8 (*CXCL8, IL8;* Hs00174103_m1), interleukin 10 (*IL10;* Hs00961622_m1), superoxide dismutase 2 (*SOD2;* Hs00167309_m1), glutathione peroxidase 4 (*GPX4*; Hs00989766_g1), catalase (*CAT*; Hs00156308_m1). Gene expression levels were normalized to the geometric mean of reference genes. For placenta explants, beta-2-microglobulin (*B2M;* Hs00187842_m1*)*, tyrosine 3-monooxygenase/tryptophan 5-monooxygenase activation protein zeta (*YWHAZ;* Hs01122445_g1), TATA-box binding protein (*TBP;* Hs00427620_m1), and ubiquitin C (*UBC;* Hs05002522_g1) were used. For primary trophoblast cells, normalization was performed using *UBC* (Hs05002522_g1), *YWHAZ* (Hs01122445_g1), and glyceraldehyde-3-phosphate dehydrogenase (*GAPDH*; Hs02758991_g1).

### Cytokine quantification in culture supernatants

Cytokine concentrations (IL-6, TNF-α, IL-1β, and IL-18) were quantified using high-sensitivity ELISA kits, according to the manufacturer’s protocol. Assays for IL-6, TNF-α, and IL-1β were sourced from Thermo Fisher Scientific, and the IL-18 assay was obtained from R&D Systems (Minneapolis, MN, USA). All measurements were performed in technical triplicates. The absorbance was recorded using Hidex Sense β Plus multimode plate reader (Hidex, Turku, Finland).

### Caspase-1 activity assay in placental explant supernatants

Extracellular caspase-1 activity was quantified in culture supernatants from placental explants using the Caspase-Glo® 1 Inflammasome Assay (Promega, USA) following the manufacturer’s protocol for secreted enzyme detection. Briefly, 50 µL of each clarified supernatant was transferred to a white opaque 96-well plate and mixed with an equal volume (50 µL) of reconstituted Caspase-Glo® 1 reagent. Plates were incubated for 60 min at ambient temperature on an orbital shaker to facilitate signal development. Luminescence was measured using a Hidex Sense β Plus multimode plate reader (Hidex). Background signal from reagent-only wells was subtracted from all sample readings. Signal intensity reflects the concentration of catalytically active caspase-1 released into the extracellular medium. The assay was optimized for reduced-volume conditions and performed in technical duplicates.

### Immunofluorescence and confocal microscopy

#### Antibodies and reagents

The following primary antibodies and stains were used: rabbit anti-NF-κB p65 (Abcam, ab32536; dilution 1:1000) and mouse anti-E-cadherin (Cell Signaling Technology, #14472; dilution 1:200). Secondary antibodies included Alexa Fluor™ Plus 488-conjugated goat anti-mouse IgG (H + L), highly cross-adsorbed (Invitrogen, A32723), and Alexa Fluor™ Plus 647-conjugated goat anti-rabbit IgG (H + L), highly cross-adsorbed (Invitrogen, A32733), both used at a dilution of 1:2000. ProLong™ Glass Antifade Mountant with NucBlue™ Stain was purchased from Invitrogen (P36981).

#### Assessment of NF-κB/p65 translocation by immunofluorescence staining

Cells were cultured in imaging chambers (Zell-Kontakt, Cat. No. ZLK-8014–80, Lot 502201) and treated with CBD at 31.80 µM for 24 h, followed by stimulation with LPS (0.5 µg/mL) for 40 min. Cells were then fixed with 4% paraformaldehyde (PFA) in PBS for 15 min at room temperature (RT), followed by permeabilization with 0.2% Triton X-100 in PBS for 15 min. After three washes with PBS containing 0.1% Tween-20, cells were blocked with a buffer containing 1% BSA and 10% normal goat serum (Abcam, ab7481) for 1 h at RT to reduce non-specific binding. Cells were then incubated overnight at 4 °C with primary antibodies diluted in blocking buffer, followed by three washes in PBS with 0.1% Tween-20, and a 120-min incubation at RT with the appropriate fluorophore-conjugated secondary antibodies. After final washes, coverslips were mounted using ProLong™ Glass Antifade Mountant with NucBlue™ nuclear stain (Invitrogen). Imaging was performed using a Nikon Ti-E epifluorescence microscope and an A1 + laser scanning confocal microscope equipped with × 20 and × 100 oil immersion objective lenses (Nikon, Tokyo, Japan). Image acquisition and analysis were performed using NIS-Elements AR 4.2 software (Laboratory Imaging, Hradec Králové, Czech Republic).

### Statistical analysis

qPCR, ELISA cytokines, and caspase-1 activity were analyzed using the non-parametric Kruskal–Wallis test followed by Dunn’s multiple comparisons test. Differences in NF-κB p65 nuclear localization were analyzed by one-way ANOVA followed by Dunnett’s post hoc test. All computations were performed with GraphPad Prism (GraphPad Software, San Diego, CA, USA). In the figures, significance is indicated as follows: *p ≤ 0.05, **p ≤ 0.01, ***p ≤ 0.001, and ****p ≤ 0.0001.

## Results

### CBD selectively attenuates LPS-induced pro-inflammatory gene expression in human placental explants

**Fig. 1:**
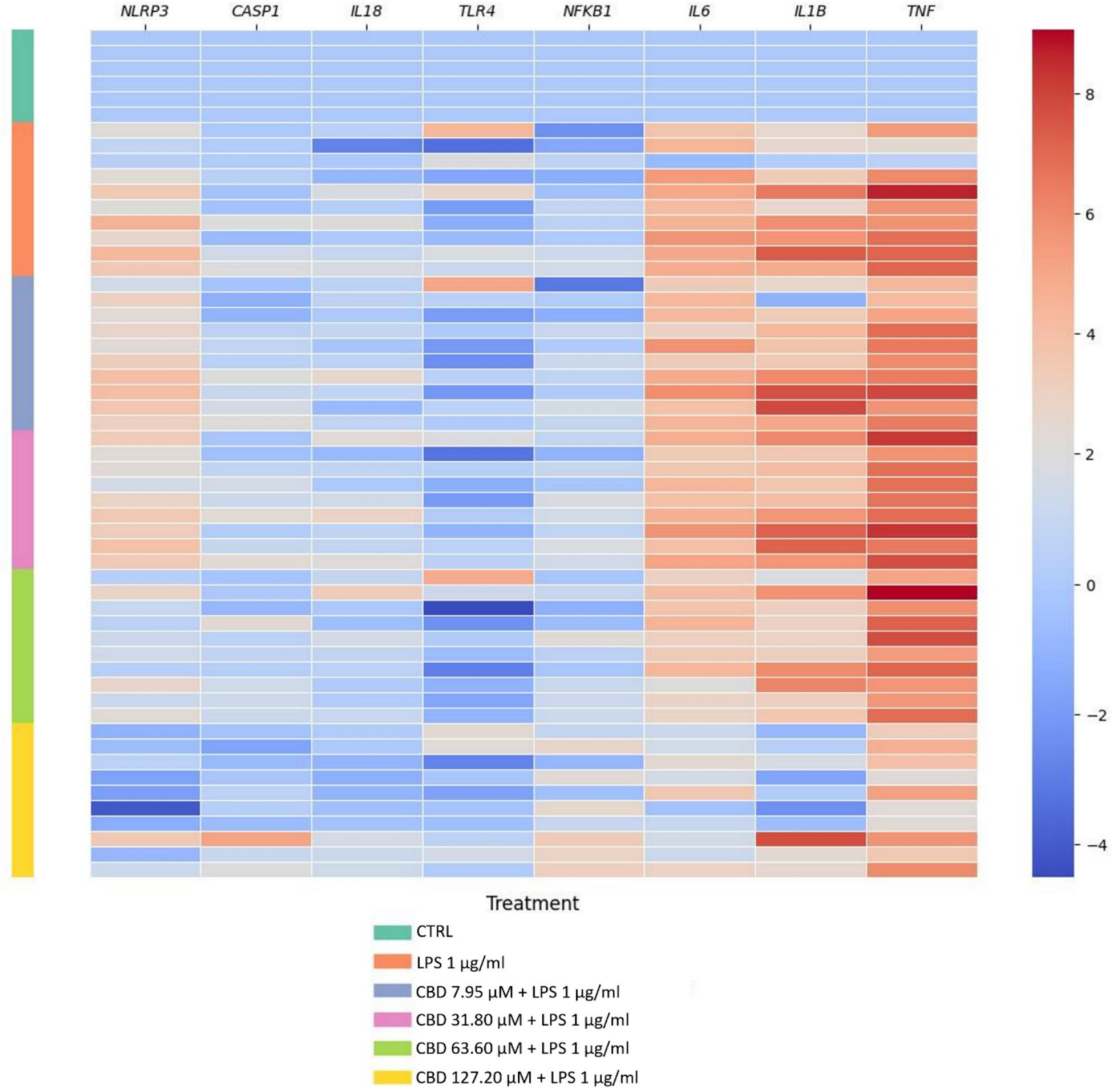
CBD modulates LPS-induced pro-inflammatory gene expression in human placental explants. Heatmap displaying log₂-transformed, row-normalized gene expression profiles of key inflammatory mediators in human placental explants pretreated with increasing concentrations of CBD (7.95–127.20 µM) for 48 h, followed by stimulation with LPS (1 µg/mL) for 4 h. Each column represents an individual gene; each row corresponds to a biological replicate. Color scale indicates relative expression (red = upregulation, blue = downregulation). Statistical analyses were performed using the non-parametric Kruskal–Wallis test followed by Dunn’s multiple-comparisons test.

We investigated transcriptional changes in an ex vivo model of placental inflammation by analyzing mRNA expression in human placental explants treated with CBD (7.95–127.20 µM) for 48 h, followed by LPS (1 µg/mL) stimulation for 4 h. As shown in Fig. 1, LPS markedly upregulated transcripts of pro-inflammatory cytokines *IL6*, *IL1B*, and *TNF*, confirming robust activation of innate immune signaling. CBD pretreatment attenuated this response in a concentration-dependent manner. At 63.60 and 127.20 µM, cytokine expression was substantially reduced, approaching levels observed in unstimulated controls. Notably, expression of *NLRP3*, a key inflammasome component, was also diminished at the highest CBD dose, suggesting partial inhibition of inflammasome priming.

In contrast, transcripts encoding upstream mediators such as TLR4, CASP1, and NFKB1 were not altered by CBD, LPS, or CBD + LPS treatment, indicating that CBD’s transcriptional effects are more prominent at the level of downstream effector cytokines, including IL-1β and IL-18.

### CBD reduces LPS-induced secretion of pro-inflammatory cytokines in human placental explants

**Fig. 2:**
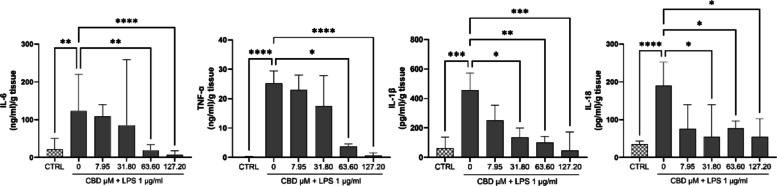
CBD suppresses LPS-induced secretion of pro-inflammatory cytokines in human placental explants. Cytokine concentrations of IL-6, TNF-α, IL-1β, and IL-18 were quantified in culture supernatants from human placental explants pretreated with CBD (7.95–127.20 µM) for 48 h, followed by stimulation with LPS (1 µg/mL) for 4 h. Data are expressed as (ng/ml)/g tissue for IL-6 and TNF-α or (pg/ml)/g tissue for IL-1β, and IL-18 (median (IQR); *n* = 10). Statistical analysis was performed using the Kruskal–Wallis test followed by Dunn’s post hoc comparisons. *p < 0.05, **p < 0.01, ***p < 0.001, ****p < 0.0001.

As shown in Fig. 2, LPS stimulation significantly increased the secretion of all four cytokines compared to untreated controls, confirming effective induction of an inflammatory response. Optimization of LPS stimulation time (4 h vs. 18 h) in both placental explants and STB cells is shown in Additional file Figure S1−2. CBD pretreatment attenuated cytokine release in a dose-dependent manner. Secretion of IL-1β and IL-18 was significantly reduced at 31.80, 63.60, and 127.20 µM CBD. TNF-α levels were significantly diminished at 63.60 and 127.20 µM*,* while IL-6 secretion was also significantly suppressed at these concentrations. These findings corroborate the transcriptional data and demonstrate that CBD exerts potent anti-inflammatory effects at the protein level in LPS-stimulated human placental explants.

### Caspase-1 activity in human placental explants following LPS and CBD treatment

Caspase-1 activity was assessed in human placental explants following LPS stimulation and CBD treatment. LPS-stimulated explants showed values comparable to controls, with a slight numerical increase that did not reach statistical significance. CBD treatment, at all tested concentrations, did not significantly alter caspase-1 activity in either LPS-stimulated or unstimulated explants.

### CBD attenuates LPS-induced pro-inflammatory gene expression in primary trophoblast cells

As shown in the heatmap (Fig. 4A), stimulation with LPS (0.5 µg/mL) triggered a robust pro-inflammatory transcriptional response in STB cells, characterized by upregulation of key cytokines (*IL1B*, *TNF*, *IL6*, *IL18*), pattern recognition receptors (*TLR4*), and inflammatory signaling mediators (*NFKB1*, *NLRP3*, *CASP1*). CBD was administered as a pretreatment prior to LPS exposure, and its effects were evaluated across a range of concentrations (0.32–31.80 µM). *IL1B* and *TNF* expression was consistently suppressed across all CBD concentrations, with maximal inhibition observed at 31.80 µM. *IL6* also showed a concentration-dependent decline, while IL18, a downstream inflammasome-derived cytokine, showed more variability but followed a similar downward trend.

In contrast, the expression of upstream mediators such as *TLR4* and *NFKB1* was only partially attenuated at higher CBD concentrations, and no clear dose–response pattern was evident. Inflammasome-associated transcripts (*NLRP3* and *CASP1*) were modestly induced by LPS and remained largely unaffected by CBD pretreatment, suggesting minimal involvement of this axis in CBD-mediated immunomodulation. To determine whether these effects were mediated via canonical cannabinoid or vanilloid receptors, STB cells were co-treated with CBD and selective pharmacological antagonists: rimonabant (CB1 inverse agonist, 10 µM; Fig. 4B), SR144528 (CB2 antagonist, 10 µM; Fig. 4C), or SB366791 (TRPV1 antagonist, 10 µM; Fig. 4D). The transcriptional suppression of *IL1B*, *TNF*, and *IL6* persisted across all receptor inhibition conditions, and expression of upstream mediators remained unchanged. These findings indicate that CBD suppresses LPS-induced pro-inflammatory signaling in STB cells independently of CB1, CB2, and TRPV1: the contribution of other ECS-related targets, such as TRPV2–4, GPR55, GPR3/6/12 cannot be excluded (Rezende et al. 2023).

### CBD suppresses inflammatory cytokine secretion in trophoblasts independently of CB1, CB2, and TRPV1 receptors

To assess whether the anti-inflammatory effects of CBD in human trophoblasts involve classical cannabinoid or vanilloid receptors, STB-stage primary trophoblasts were pretreated with selective antagonists of CB1 (rimonabant, 10 µM), CB2 (SR144528, 10 µM), or TRPV1 (SB366791, 10 µM), followed by CBD administration (0.32–31.80 µM) for 24 h and subsequent stimulation with LPS (0.5 µg/mL) for 4 h. As shown in Fig. 5, CBD significantly reduced the secretion of IL-6, TNF-α, and IL-1β in a concentration-dependent manner under all conditions tested. Maximal suppression of IL-6 and TNF-α was observed at 31.80 µM, while IL-1β secretion showed a more modest but consistent reduction across concentrations. Notably, the presence of CB1, CB2, or TRPV1 antagonists did not reverse the inhibitory effect of CBD on cytokine secretion. Across all receptor-blockade conditions, cytokine levels in CBD-treated groups remained significantly lower than in LPS-only controls (*p* < 0.05 or lower), with no statistically significant differences between CBD alone and CBD + antagonist groups (*p* > 0.05). These findings indicate that the anti-inflammatory effects of CBD in STB cells persist despite CB1, CB2, or TRPV1 blockade, pointing to alternative, non-canonical pathways of action.

### CBD reduces NF-κB p65 nuclear translocation in LPS-stimulated trophoblasts

Building on these observations, we evaluated the effect of CBD on the NF-κB signaling pathway, specifically focusing on nuclear translocation of the p65 subunit. Representative immunofluorescence images (Fig. 6A) demonstrated that control cells exhibited mainly cytoplasmic localization of NF-κB p65, consistent with an unstimulated, non-inflammatory state. Upon LPS stimulation, there was a pronounced increase in nuclear localization of NF-κB p65, indicating activation of the inflammatory signaling pathway. Notably, pre-treatment with CBD significantly reduced nuclear accumulation of NF-κB p65, closely resembling the control cells, suggesting reduced NF-κB activation in response to LPS. Quantitative analysis of the NF-κB p65/DAPI nuclear intensity ratio from five independent experiments (*n* = 5) further supported these visual findings (Fig. 6B). Statistical analysis showed a significant reduction in nuclear NF-κB p65 levels following CBD treatment compared with LPS alone (*p* < 0.01). All microscopy images were captured under standardized conditions with uniform laser power, gain, and exposure settings, and randomized field selection was used to minimize potential observational bias. Collectively, these results highlight CBD's ability to effectively attenuate NF-κB signaling in primary trophoblasts, underscoring its potential anti-inflammatory properties.

## Discussion

The maternal–fetal interface represents a unique immunological environment where pro-inflammatory signals must be tightly regulated to support fetal development without compromising host defense (Megli and Coyne 2022). Dysregulated placental inflammation contributes to pregnancy complications, yet targeted therapies remain elusive. While CBD has emerged as a candidate anti-inflammatory compound in other systems (Aziz et al. 2023), its role in the maternal–fetal immune environment remains poorly characterized. To address this, we employed two complementary models—term placental explants and primary syncytiotrophoblast cells—to investigate how CBD modulates innate immune responses under LPS-induced inflammatory stress. These systems enabled us to interrogate both tissue-level and cell-specific mechanisms of CBD action, revealing potential pathways through which cannabinoid signaling intersects with placental immune regulation.

This contrasts with findings in THP-1 macrophages and bronchial epithelial cells, where CBD and THC significantly suppressed NLRP3, pro-caspase-1, and IL-1β expression, particularly under LPS + ATP dual-stimulation, which fully activates the inflammasome complex (Suryavanshi et al. 2022). Similar effects were reported in LPS-challenged BV2 microglial cells, where CBD reduced NLRP3 and caspase-1 levels via CB2 and PPARγ pathways, and receptor antagonism reversed these effects, supporting a receptor-mediated mechanism (Rodrigues et al. 2024). In contrast, our model employed LPS-only stimulation to better reflect maternal immune activation, a physiologically relevant condition in which bacterial endotoxins, such as LPS, are sufficient to trigger placental inflammation, without the need for additional sterile danger signals like extracellular ATP (Moss et al. 2025). The addition of ATP, although common in monocultures, can be cytotoxic in complex tissues and may lead to artificial overactivation, potentially obscuring moderate pharmacological responses (Di Virgilio et al. 2023).

Consistent with gene expression data, CBD pretreatment significantly reduced LPS-induced secretion of IL-6, TNF-α, IL-1β, and IL-18 in human placental explants in a concentration-dependent manner (Fig. 2). IL-1β and IL-18 were suppressed at ≥ 31.80 µM, whereas IL-6 and TNF-α required higher concentrations (63.60–127.20 µM), indicating differential cytokine sensitivity. Although CBD had minimal effects on NLRP3 or CASP1 mRNA levels, the marked reduction in IL-1β and IL-18 protein levels suggests post-transcriptional modulation of inflammasome-related cytokine release. Similar findings have been reported in BV2 microglia, where CBD inhibited NLRP3 and caspase-1 activity via CB2 and PPARγ signaling (Rodrigues et al. 2024), and in THP-1 macrophages, where CBD reduced IL-1β and IL-18 secretion without consistent changes in gene expression (Suryavanshi et al. 2022).

**Fig. 3:**
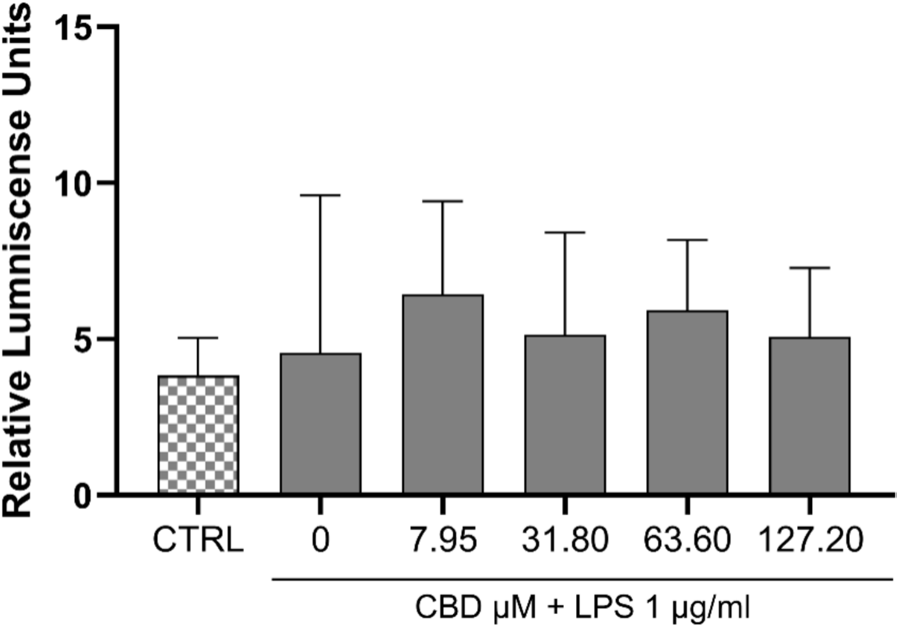
Caspase activity in human placental explants co-treated with LPS and CBD. Relative caspase-1 activity was quantified in placental explants pretreated with CBD (7.95–127.20 µM) for 48 h, followed by stimulation with LPS (1 µg/mL) for 4 h. Data are presented as median (IQR) (*n* = 6). Statistical analysis was conducted using the Kruskal–Wallis test followed by Dunn’s multiple comparisons post hoc test.

The limited suppression of NLRP3 at lower CBD concentrations may reflect tissue-specific regulation and the absence of a secondary activation signal required for full inflammasome engagement. To evaluate functional inflammasome activity, we assessed caspase-1 enzymatic function in LPS-stimulated placental explants (Fig. 3). LPS did not significantly alter caspase-1 activity, and CBD pretreatment showed no measurable effect. This pattern suggests that the reduction in IL-1β and IL-18 secretion is unlikely to result from direct caspase-1 inhibition, but may instead reflect CBD activity upstream of, or parallel to, caspase-1, consistent with prior studies in human immune and epithelial cells showing cytokine suppression via modulation of NF-κB and MAPK signaling rather than inflammasome silencing (Martinez Naya et al. 2024; Sermet et al. 2021). This proteo-genomic signature reveals that CBD fine-tunes placental immune responses by acting at both transcriptional and post-transcriptional levels, selectively attenuating cytokine pathways relevant to maternal–fetal immune regulation.

**Fig. 4:**
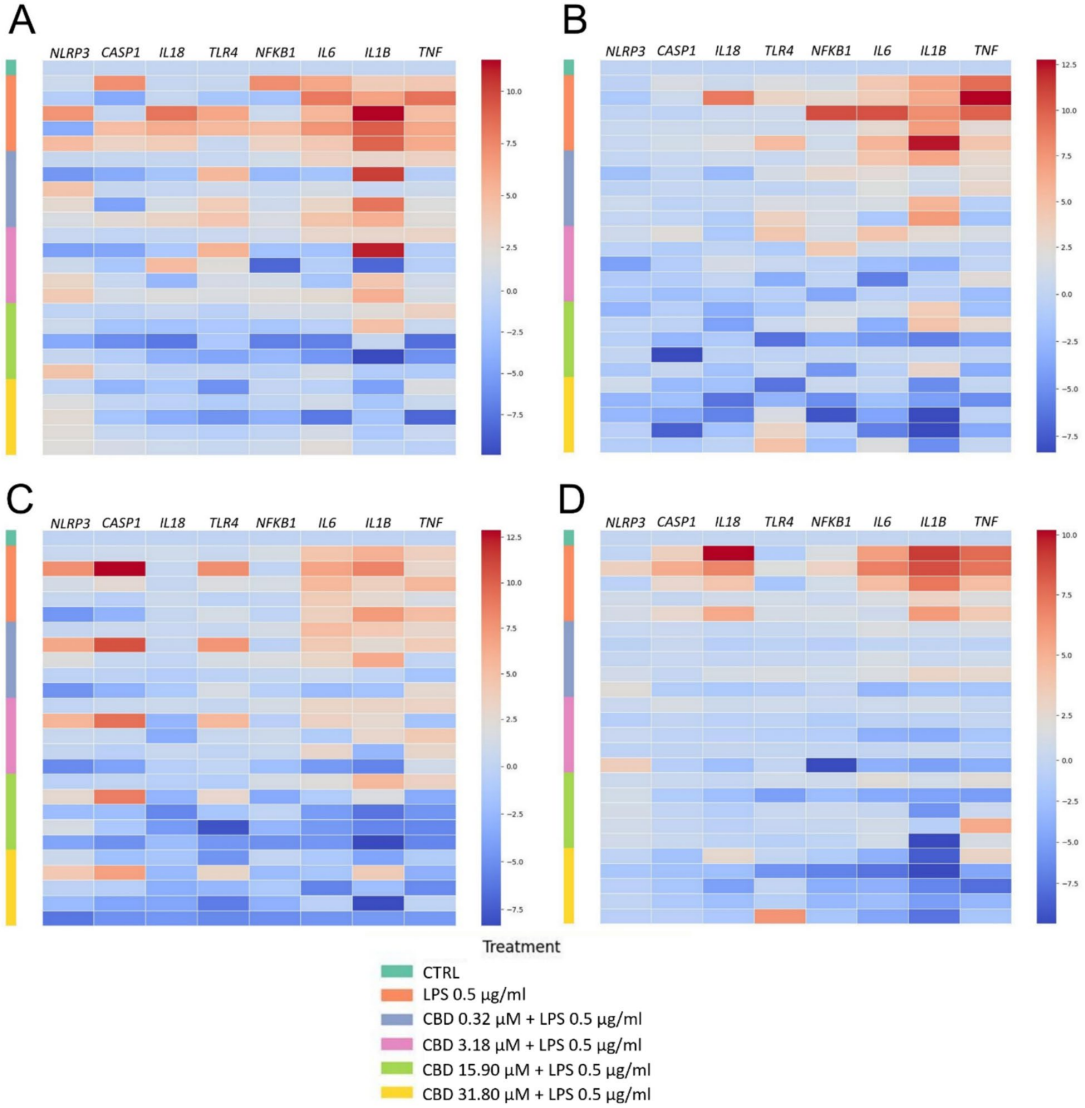
Heatmaps of normalized gene expression in STB cells exposed to LPS, CBD, and receptor antagonists. Primary human trophoblasts differentiated to the STB stage were pretreated with CBD (0.32–31.80 µM) for 24 h prior to stimulation with LPS (0.5 µg/mL) for 4 h. The heatmap shows log-transformed, normalized gene expression of pro-inflammatory cytokines (*IL1B*, *TNF*, *IL6*, *IL18*), upstream signaling molecules (*TLR4*, *NFKB1*), and inflammasome-related genes (*NLRP3*, *CASP1*). Experimental conditions included no antagonist (**A**), CB1 antagonist rimonabant (**B**), CB2 antagonist SR144528 (**C**), and TRPV1 SB366791 (**D**). Downregulated transcripts are shown in blue; upregulated transcripts in red. LPS-induced cytokine expression was attenuated by CBD in a concentration-dependent manner, with minimal changes observed in inflammasome components. The anti-inflammatory effect of CBD persisted in the presence of CB1, CB2, and TRPV1 antagonists, indicating that its action does not rely on these canonical receptors. Statistical analyses were performed using the non-parametric Kruskal–Wallis test followed by Dunn’s multiple-comparisons test.

Given the cellular heterogeneity of placental explants, we evaluated gene expression in primary STB-stage trophoblasts to determine whether these cells contribute to the anti-inflammatory effects of CBD observed in the explant model (Fig. 4). Consistent with explant results, CBD dose-dependently suppressed LPS-induced transcription of *IL1B*, *IL6*, *IL18*, and *TNF*, with maximal effects at 15.90–31.80 µM. Notably, CBD also partially downregulated *TLR4* and *NFKB1* transcripts, suggesting early interference in innate immune signaling. This effect may reflect greater accessibility of CBD in monolayer cultures compared to explants, where complex tissue structure can limit diffusion. *CASP1* mRNA remained unchanged, indicating that CBD’s modulation of *IL-1B* and *IL18* gene expression does not involve direct inhibition of inflammasome transcription. Parallel findings in other cell types support this upstream mechanism. In human periodontal ligament cells, CBD inhibited LPS-induced TLR4 expression and NF-κB activation, leading to reduced IL-6 and TNF-α secretion (Chen et al. 2023). Additionally, in coronary artery endothelial cells, CBD blocked LPS-induced IKKβ phosphorylation and NF-κB activation (Blessing et al. 2024). Together, these data suggest that CBD may exert anti-inflammatory effects in trophoblasts by modulating both receptor-level signaling (TLR4–NF-κB) and downstream cytokine transcription, reinforcing its potential as a selective immunomodulator in placental tissue. In contrast, CBD did not significantly alter expression of anti-inflammatory cytokines (*IL8, IL10*) or antioxidant enzymes (*SOD2, CAT, GPX1*) in placenta explants, despite their reported roles in modulating placental inflammation and redox-sensitive immune signaling (Vornic et al. 2024; Chatterjee et al. 2014) (Additional file Figure S3).

To elucidate the mechanism underlying CBD’s anti-inflammatory effects in placental cells, we first investigated the potential involvement of canonical cannabinoid receptors (CB1 and CB2) and the vanilloid receptor TRPV1. Although CBD exhibits low affinity for the orthosteric binding sites of these receptors, accumulating evidence shows that it can modulate their activity through non-classical interactions—acting, for example, as a negative allosteric modulator at CB1, an inverse agonist at CB2, and an agonist at TRPV1 (Laprairie et al. 2015; Carruthers and Grimsey 2024; Etemad et al. 2022). These atypical modes of receptor engagement have been implicated in modulating inflammatory responses across multiple systems. In light of this, and considering that CB1, CB2, and TRPV1 are expressed in human placental tissue and have been linked to local immune regulation (Rokeby et al. 2023), it was biologically plausible to test their functional contribution to CBD’s effects in this context.

**Fig. 5:**
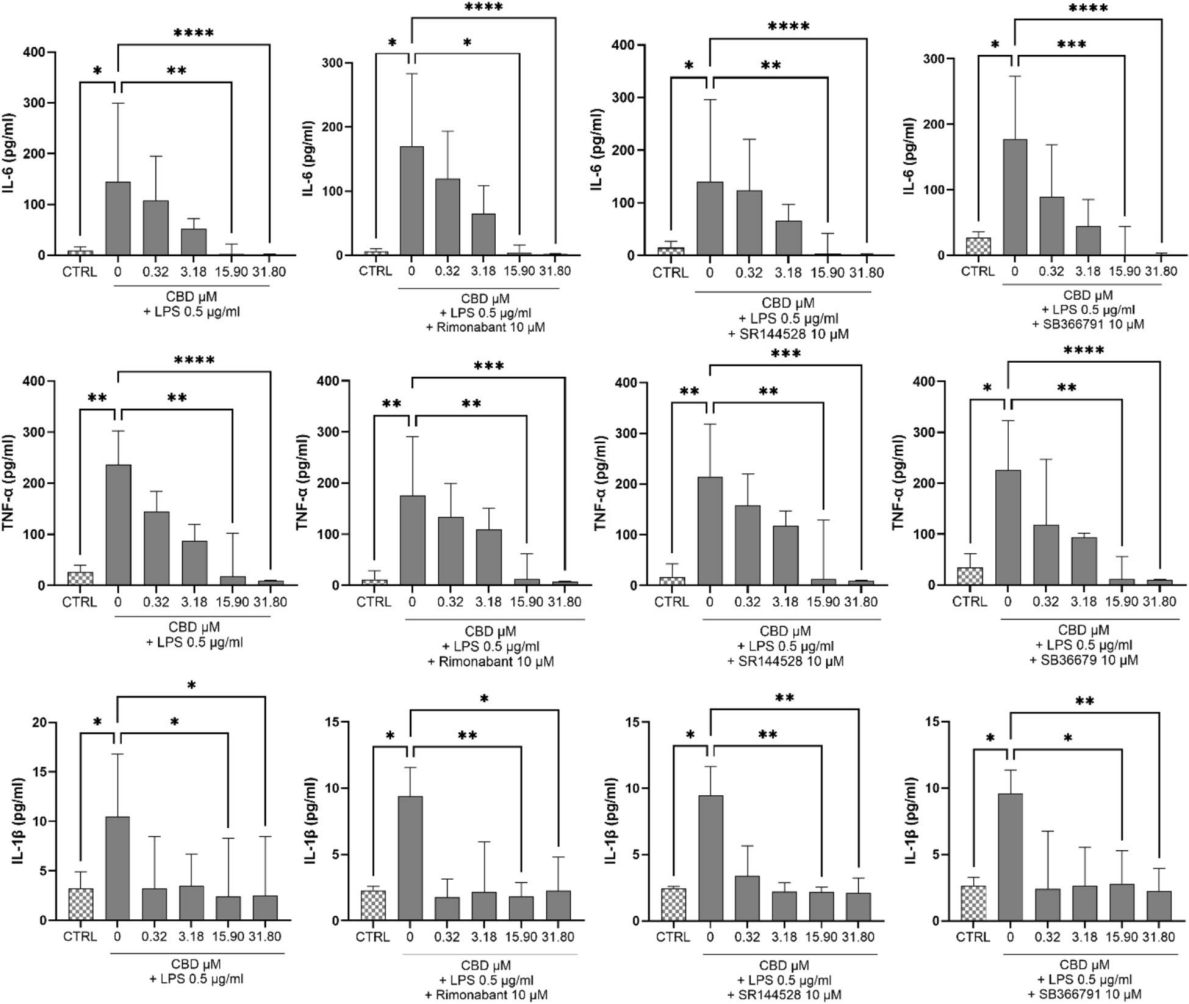
CBD-mediated IL-6 and TNF-α suppression in LPS-stimulated trophoblasts persists despite CB1, CB2, or TRPV1 antagonism. Primary human trophoblasts differentiated to the STB stage were pretreated with CBD (0.32–31.80 µM) alone or in combination with selective antagonists of CB1 (rimonabant, 10 µM), CB2 (SR144528, 10 µM), or TRPV1 (SB366791, 10 µM) for 24 h, followed by stimulation with LPS (0.5 µg/mL) for 4 h. CBD significantly reduced the secretion of IL-6 (top panels), TNF-α (middle panels), and IL-1β (bottom panels) in a concentration-dependent manner. Co-treatment with receptor antagonists did not abrogate the suppressive effect of CBD, indicating that its anti-inflammatory action is independent of CB1, CB2, and TRPV1 signaling. Data are presented as median (IQR) from six independent placentas (*n* = 6). Statistical analysis was performed using the Kruskal–Wallis test followed by Dunn’s post hoc test. *p < 0.05, **p < 0.01, ***p < 0.001, ****p < 0.0001; ns = not significant.

In our study, selective antagonists for CB1 (rimonabant), CB2 (SR144528), and TRPV1 (SB366791) failed to reverse CBD-induced suppression of inflammatory cytokines and gene expression in LPS-stimulated trophoblasts, indicating that the anti-inflammatory action of CBD in placental tissue is independent of these canonical receptors (Figs. 4 and 5). This aligns with prior findings, including studies showing that cannabinoids can exert antioxidant and cell-survival effects, as well as modulate neuronal activity and cancer-related processes, independently of known cannabinoid receptors (Chen and Buck 2000; Soderstrom et al. 2017). Nevertheless, other reports have shown that CBD can directly activate, desensitize TRPV1 and TRPV2 channels (Iannotti et al. 2014) and exert receptor-mediated effects depending on the system and context (Oz et al. 2022). Thus, our rationale for pharmacologically probing these receptors was grounded in the biological variability of CBD’s mechanisms of action, which may be context-dependent and influenced by tissue type, receptor expression, and disease state. Together, the findings suggest that CBD attenuates inflammatory responses in placental tissue by modulating downstream signaling nodes, including NF-κB, rather than engaging the endocannabinoid receptors most commonly studied to date.

**Fig. 6:**
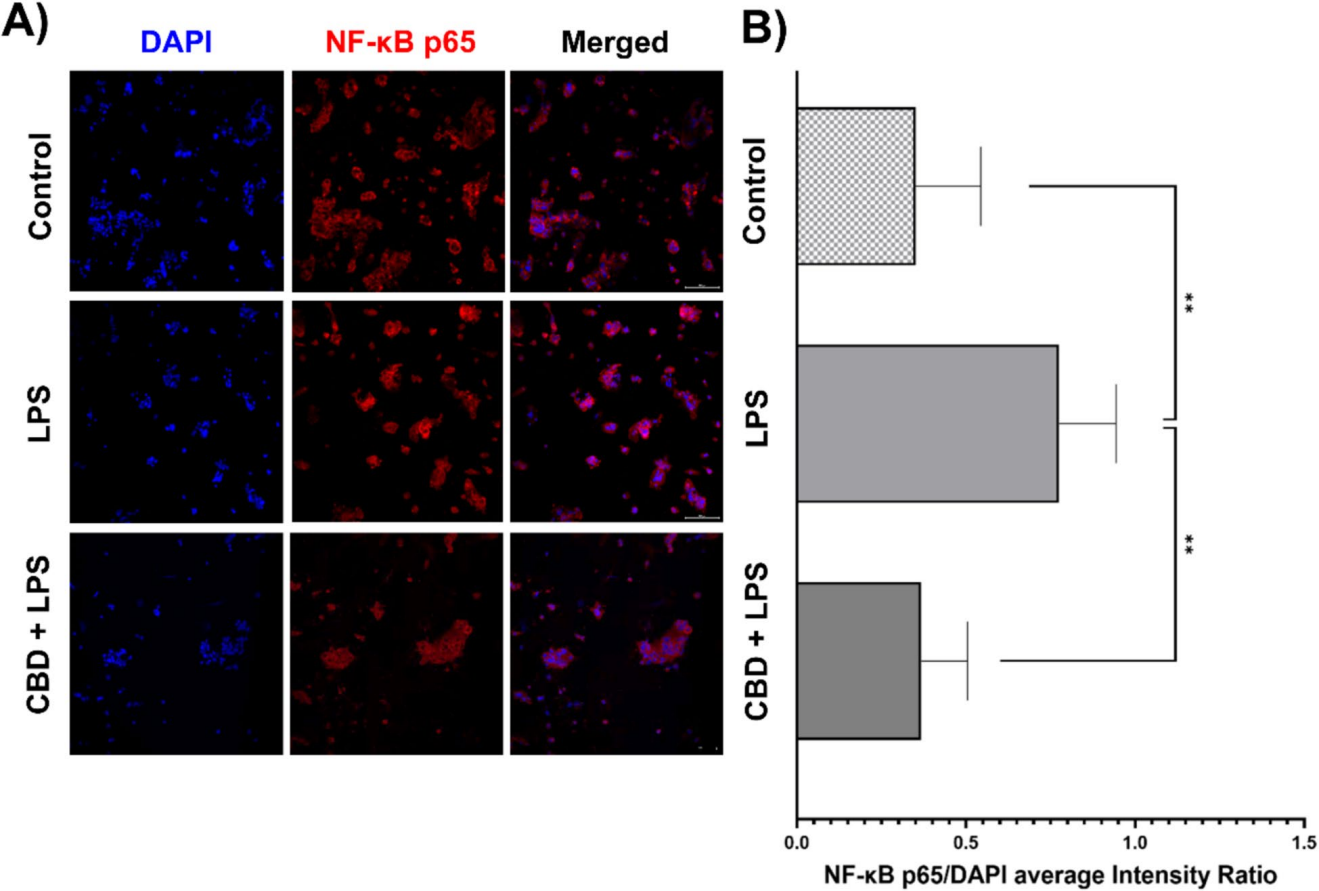
CBD modulates NF-κB p65 nuclear translocation in primary trophoblasts upon LPS stimulation. Representative confocal immunofluorescence images and intensity profile analysis of NF-κB p65 (red) and DAPI (blue, nuclear stain) in human primary cytotrophoblasts under different treatment conditions. **A** Representative confocal immunofluorescence images of primary human cytotrophoblasts showing DAPI (blue, nuclear stain), NF-κB p65 (red), and merged channels under three treatment conditions: untreated control (media only), 0.5 μg/ml LPS stimulation for 40 min, and CBD pre-treatment (31.80 μM, 24 h) followed by LPS (0.5 μg/ml, 40 min). Images illustrate the redistribution of NF-κB p65 from the cytoplasm to the nucleus upon LPS stimulation and its attenuation by CBD. **B** Quantification of the nuclear NF-κB p65 intensity relative to DAPI (NF-κB p65/DAPI ratio) across treatment groups. Data represent mean ± SD from *n* = 5 independent experiments. Statistical significance was assessed using one-way ANOVA followed by Dunnett’s multiple comparisons test versus the LPS group. *p < 0.05, **p < 0.01, ***p < 0.001. Scale bar = 100 μm. Magnification: 20 ×

Following the exclusion of canonical inflammasome regulation and receptor-mediated pathways, we investigated NF-κB nuclear translocation as a potential mechanism underlying CBD’s anti-inflammatory effects in placental cells. NF-κB plays a central role in regulating pro-inflammatory gene expression, and its activation involves cytoplasmic-to-nuclear translocation of the p65 subunit. Confocal imaging of primary human trophoblasts showed that LPS stimulation was associated with increased nuclear translocation of NF-κB p65. This effect was significantly attenuated by CBD pretreatment (31.80 µM), as shown by reduced nuclear p65 signal relative to DAPI (Fig. 6), indicating attenuation of NF-κB activation. These findings are consistent with previous studies in other human cell types. In coronary artery endothelial cells, CBD suppressed LPS-induced IKKβ phosphorylation and prevented nuclear translocation of NF-κB p65, resulting in reduced expression of inflammatory mediators (Teichmann et al. 2023). Similarly, in human periodontal ligament cells, CBD inhibited TLR4 expression and NF-κB phosphorylation in response to LPS, leading to reduced IL-6 and TNF-α secretion (Chen et al. 2023). While these studies used biochemical and Western blot techniques, our results extend the evidence using direct spatial analysis through confocal microscopy in trophoblasts. Furthermore, the involvement of TLR4–MyD88 signaling in LPS-induced NF-κB activation has been demonstrated in macrophage models, in which MyD88 deficiency abrogated nuclear translocation of p65 (Sakai et al. 2017). Our data, showing reduced NF-κB nuclear accumulation alongside partial TLR4 transcript suppression, suggest a similar pathway may operate in trophoblasts and be modulated by CBD.

These findings highlight the broader significance of NF-κB modulation in placental immunity. Beyond its canonical role in inflammatory gene regulation, NF-κB functions as a central integrator of immune signaling, cell survival, and metabolic adaptation (Liu et al. 2017; Guo et al. 2024). During pregnancy, its activity is tightly regulated across gestation: transient activation supports implantation and labor, while mid-gestational suppression maintains maternal–fetal tolerance. Disruption of this temporal control—via infection, autoimmunity, or pharmacological agents—has been implicated in pregnancy complications, including preeclampsia, preterm labor, and intrauterine growth restriction (IUGR) (Gomez-Chavez et al. 2021). Impaired downregulation of NF-κB p65 in maternal T cells is associated with enhanced Th1/Th17 polarization and elevated pro-inflammatory cytokines in IUGR. Although systemic NF-κB inhibition (e.g., IKKβ or proteasome targeting) has proven clinically unfeasible due to severe toxicities, CBD offers a potentially safer approach by attenuating NF-κB activation without complete pathway suppression (Ariyakumar et al. 2021). Nonetheless, due to NF-κB’s pleiotropic functions and its integration within a broader transcriptional network involving STAT3 and AP-1, indiscriminate inhibition may disrupt essential immunological adaptations of pregnancy (Ji et al. 2019). Therapeutic strategies must therefore account for the spatial and temporal specificity of NF-κB signaling in gestation. These insights emphasize that any modulation—natural or synthetic—must respect the temporal and spatial complexity of NF-κB signaling in pregnancy. In addition, CBD exhibits pleiotropic pharmacology and can engage multiple signaling nodes beyond those interrogated in the present study. In addition, inter-individual variability in placental receptor expression and metabolic capacity may influence the magnitude of response, reinforcing the need for cautious translational interpretation.

Our findings indicate that CBD reduces placental inflammation, independent of CB1, CB2, and TRPV1 signaling, and is associated with decreased NF-κB p65 nuclear translocation. This effect was consistent across explants and primary trophoblasts, and bypassed canonical CB1, CB2, and TRPV1 signaling. While this underscores CBD’s potential as a selective immunomodulator at the maternal–fetal interface, it also highlights the need for caution when targeting immune pathways in the placenta—an organ where inflammation is not merely a pathology, but a tightly regulated developmental signal (Hauguel-de Mouzon and Guerre-Millo 2006). Immune activity at this interface governs not only host defense but fetal programming, vascular adaptation, and tolerance. Perturbing these pathways may carry latent consequences (Goldstein et al. 2020). Notably, emerging evidence indicates that CBD can also disrupt placental tryptophan metabolism—a pathway intimately linked to cytokine regulation, immune tolerance, and redox balance (Portillo et al. 2024). Such interference could further compound immune dysregulation during gestation. Thus, the pharmacological appeal of CBD must be weighed against its potential to alter developmental signaling, particularly in pregnancy. Rigorous toxicological profiling, grounded in human-relevant models and frameworks such as DOHaD, is essential.

## Supplementary Information

Supplementary Material 1.

## Data Availability

The datasets generated and/or analyzed during the current study are available from the corresponding author on reasonable request.

## Abbreviations

ANOVA: Analysis of Variance
AP-1: Activator Protein 1
ATP: Adenosine Triphosphate
B2M: Beta-2-Microglobulin
CB1: Cannabinoid Receptor Type 1
CB2: Cannabinoid Receptor Type 2
CAT: Catalase
CASP1: Caspase 1
cDNA: Complementary DNA
CXCL8, IL-8: C-X-C Motif Chemokine Ligand 8, Interleukin 8
DAPI: 4′,6-Diamidino-2-Phenylindole
DMEM: Dulbecco’s Modified Eagle Medium
DOHaD: Developmental Origins of Health and Disease
DMSO: Dimethyl Sulfoxide
ELISA: Enzyme-Linked Immunosorbent Assay
ECS: Endocannabinoid System
FBS: Fetal Bovine Serum
GAPDH: Glyceraldehyde-3-Phosphate Dehydrogenase
GPX4: Glutathione Peroxidase 4
HPLC: High-Performance Liquid Chromatography
IL: Interleukin (e.g., IL-1β, IL-6, IL-10, IL-18)
IUGR: Intrauterine Growth Restriction
LPS: Lipopolysaccharide
MAPK: Mitogen-Activated Protein Kinase
mRNA: Messenger RNA
NF-κB: Nuclear Factor Kappa-Light-Chain-Enhancer of Activated B Cells
NFKB1: Nuclear Factor Kappa B Subunit 1
NLRP3: NOD-Like Receptor Family, Pyrin Domain Containing 3
PBS: Phosphate-Buffered Saline
PCR: Polymerase Chain Reaction
PFA: Paraformaldehyde
PPARG: Peroxisome Proliferator-Activated Receptor Gamma
qPCR: Quantitative Polymerase Chain Reaction
RNA: Ribonucleic Acid
RT: Room Temperature
RT-qPCR: Reverse Transcription Quantitative Polymerase Chain Reaction
SD: Standard Deviation
SOD2: Superoxide Dismutase 2
STB: Syncytiotrophoblast
TBP: TATA-Box Binding Protein
TLR4: Toll-Like Receptor 4
TNF-α: Tumor Necrosis Factor Alpha
TRPV1: Transient Receptor Potential Vanilloid 1
UBC: Ubiquitin C
YWHAZ: Tyrosine 3-Monooxygenase/Tryptophan 5-Monooxygenase Activation Protein Zeta
