# Supplementary material for: Cannabidiol reduces LPS-induced inflammatory response in the human placenta by reducing NF-κB translocation
Source: J Cannabis Res. 2025 Dec 6;8:8. doi: 10.1186/s42238-025-00369-6 (PMC12797442; doi:10.1186/s42238-025-00369-6)
Supplement: Supplementary file 1 — Supplementary Material 1. [file 42238_2025_369_MOESM1_ESM.docx]

**Supplementary data**

**Cannabidiol reduces LPS-induced inflammatory response in the human placenta by blocking** **NF-κB translocation**

**Cytokine release in the supernatant media:**


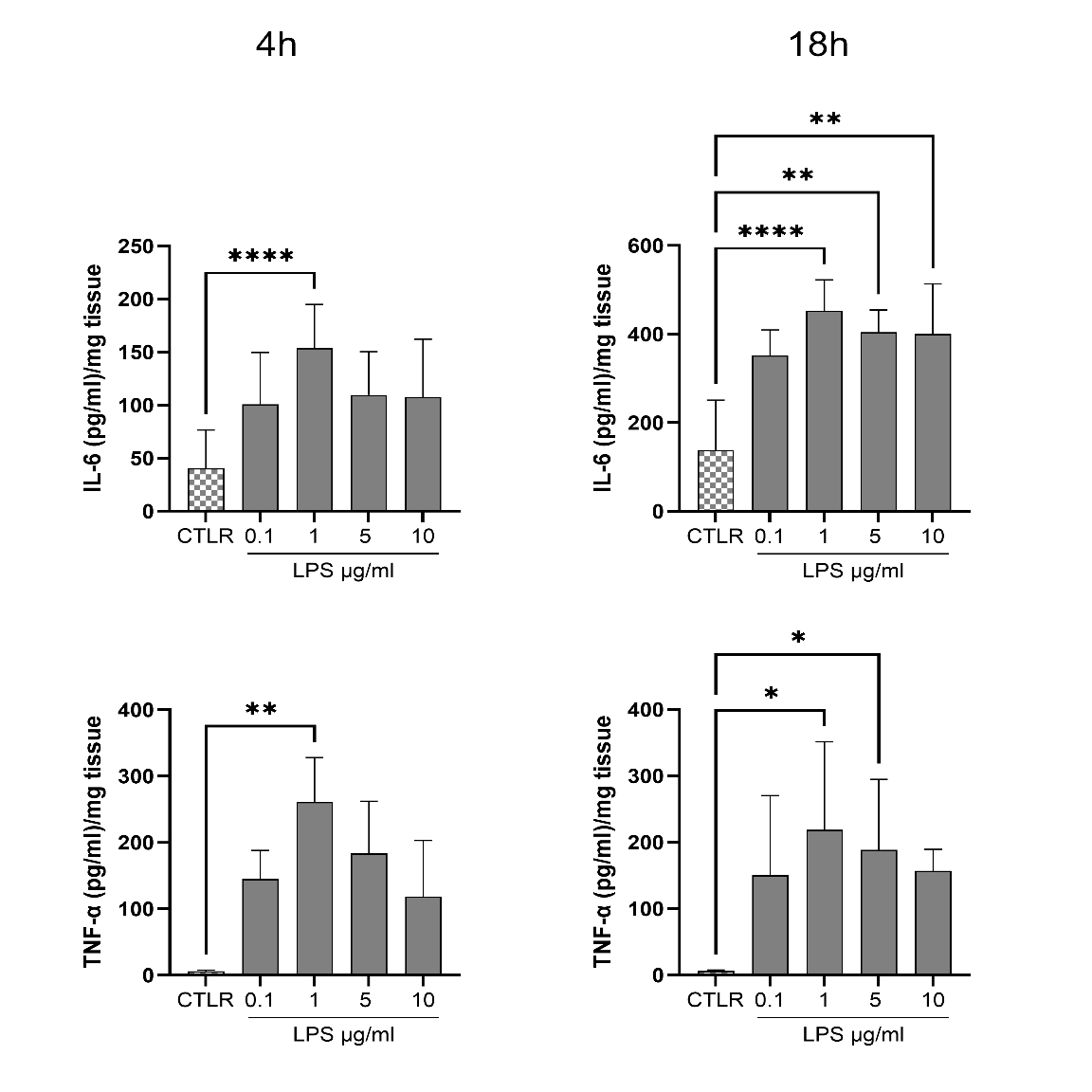


**Figure S1.** *Human placenta explants incubated for two different durations (4 and 18 hours) with varying concentrations of LPS.* Data are presented as mean with SD; n = 8 for IL-6 and n=4 for TNF-α. Statistical significance was evaluated using the non-parametric Kruskal–Wallis test, followed by Dunn's multiple comparisons test; * (p ≤ 0.05), ** (p ≤ 0.01) and ****(p ≤ 0.0001).


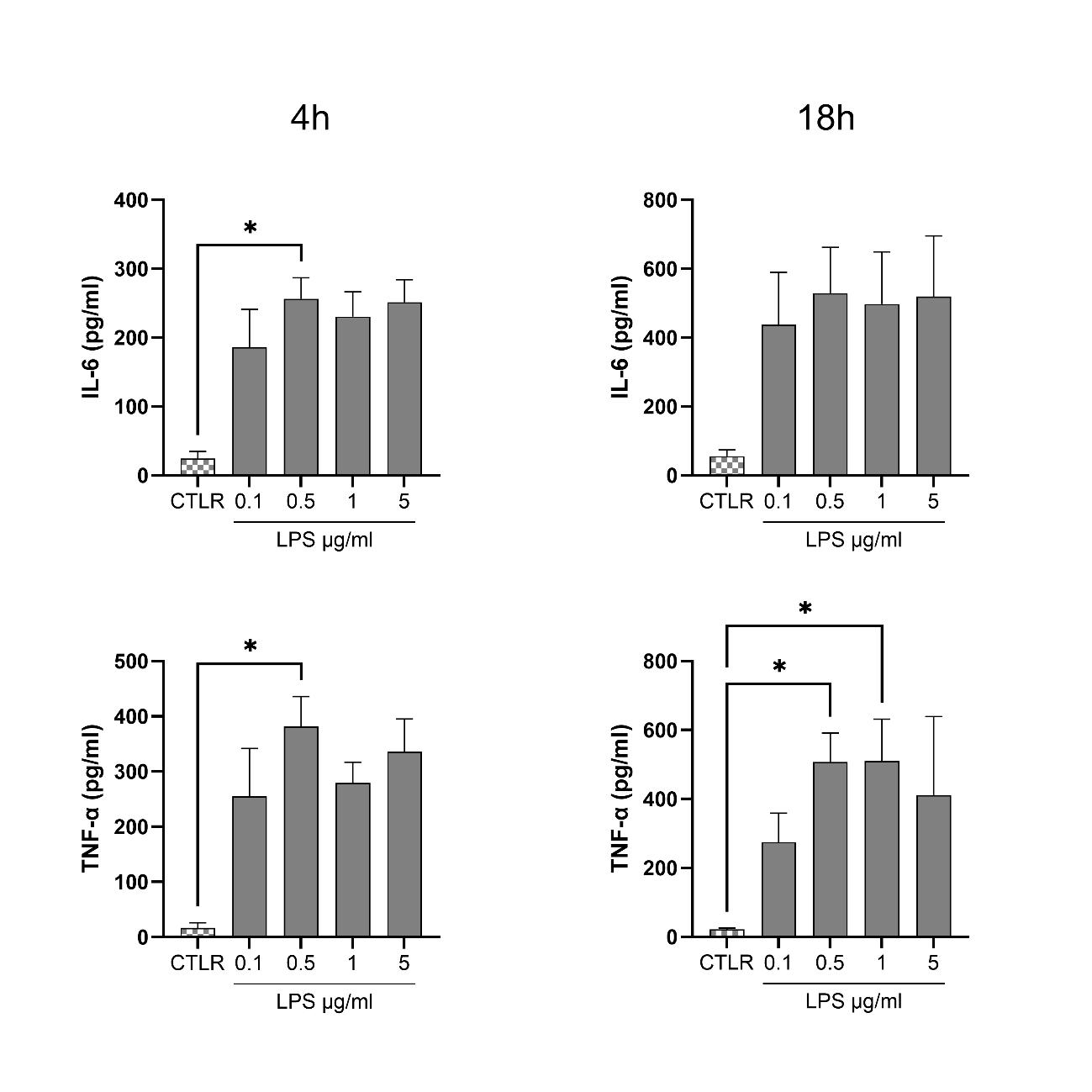
**Figure S2.** *Syncytiotrophoblast cells incubated for two different durations (4 and 18 hours) with varying concentrations of LPS.* Data are presented as mean with SD; n = 3. Statistical significance was evaluated using the non-parametric Kruskal–Wallis test, followed by Dunn's multiple comparisons test; * (p ≤ 0.05).


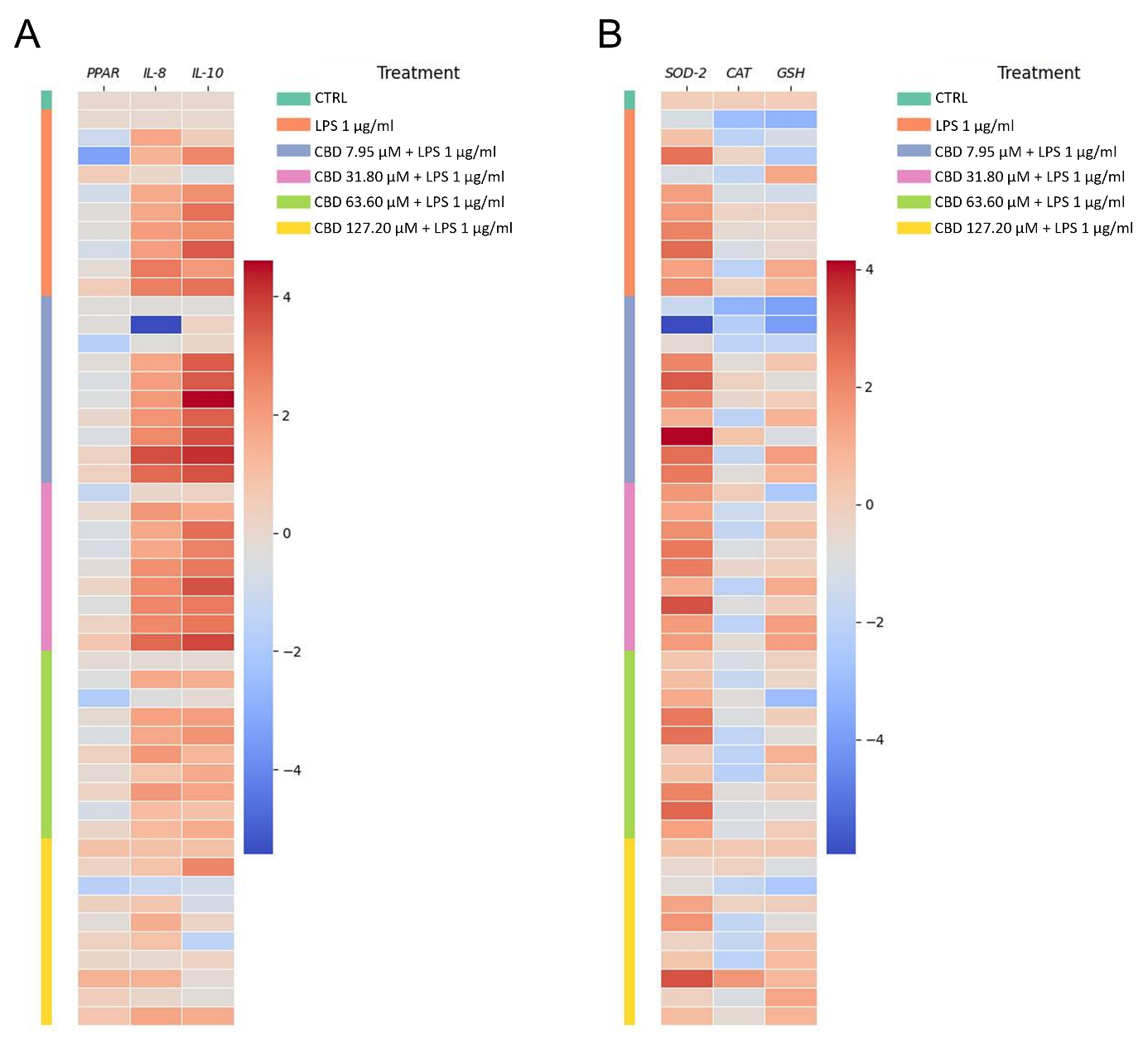


**Figure S3.** *Heatmaps of normalized gene expression in placental explants exposed to LPS and CBD****.*** *(A) PPARG and anti-inflammatory cytokines. (B) Antioxidant enzymes.*

The heatmaps present log₂-transformed, row-normalized gene expression profiles in human placental explants pretreated with increasing concentrations of CBD (7.95–127.20 µM) for 48 hours, followed by stimulation with LPS (1 µg/mL) for 4 hours. Each column represents a specific gene, and each row corresponds to a biological replicate. The color scale indicates relative expression levels, with red representing upregulation and blue representing downregulation.
